# The Role of Illness Perceptions, Coping, and Self-Efficacy on Adherence to Precautionary Measures for COVID-19

**DOI:** 10.3390/ijerph17186540

**Published:** 2020-09-08

**Authors:** Yuen Yu Chong, Wai Tong Chien, Ho Yu Cheng, Ka Ming Chow, Angelos P. Kassianos, Maria Karekla, Andrew Gloster

**Affiliations:** 1 The Nethersole School of Nursing, Faculty of Medicine, The Chinese University of Hong Kong, Hong Kong, China; wtchien@cuhk.edu.hk (W.T.C.); hycheng@cuhk.edu.hk (H.Y.C.); kmchow@cuhk.edu.hk (K.M.C.); 2 Department of Psychology, University of Cyprus, P.O. Box 20537, Nicosia 1678, Cyprus; angelos.kassianos@ucl.ac.uk (A.P.K.); mkarekla@ucy.ac.cy (M.K.); 3 Department of Applied Health Research, University College London, London WC1E 6BT, UK; 4 Department of Psychology, Division of Clinical Psychology and Intervention Science, University of Basel, 4001 Basel, Switzerland; andrew.gloster@unibas.ch

**Keywords:** COVID-19, adherence, public health, avoidance, coping

## Abstract

As the novel coronavirus disease 2019 (COVID-19) pandemic continues, engaging the public in adherence to precautionary measures for preventing COVID-19 spread or infection becomes difficult. The present study aims to extend our understanding of how illness perceptions, coping, and self-efficacy affect adherence to precautionary measures among the public. An online survey was administered between April and June 2020 to a sample of 514 Hong Kong citizens. Variables considered were illness perceptions toward COVID-19, problem-solving, avoidance-based coping, self-efficacy, as well as adherence to precautionary measures including physical distancing, limiting unnecessary travelling, and washing hands regularly with soap and water. Adjusted structural equation model showed that illness perceptions toward COVID-19 had significant direct effect on their adherence to precautionary measures (unstandardized β = 0.50, [95% CI, 0.28, 0.80], *p* = 0.001), and indirect effects through avoidance-based coping (β = −0.10 [95% CI, −0.26, −0.01], *p* = 0.016) and self-efficacy (β = −0.10, [95% CI, −0.18, −0.01], *p* = 0.025). These results imply that apart from emphasizing the health hazards of a novel infectious disease, an effective public health intervention and crisis communication should address avoidance-based coping and self-efficacy of the public in adherence to precautionary measures for COVID-19.

## 1. Introduction

The novel coronavirus disease 2019 (COVID-19) outbreak began in China on December 2019, with more than 20 million confirmed cases and 735,000 deaths worldwide as of 11 August 2020. In view of the absence of vaccines or effective treatments, current global efforts are largely focused on containing the virus spread by implementing different precautionary measures, including improved hygiene, physical distancing, wearing facemasks, comprehensive contact tracing, and quarantining, as well as border controls. Recent epidemiological studies have found the aforementioned measures can reduce the daily effective production number (*R*_1_) for COVID-19 to less than one and the mean positively rate of COVID-19 to 0.49% per day [1,2].

It is well understood that for the precautionary measures of COVID-19 to obtain the intended effects, the public should have at least a good understanding and acceptance of the measures [3]. However, there is a growing concern among the public that the requisite behavioral changes for preventing COVID-19 spread or infection are becoming more permanent and disruptive to daily life. More importantly, there could possibly be an exponential growth of COVID-19 cases if variations of adherence to precautionary measures and ambiguity emerge across different population groups and contexts. Hong Kong is currently facing the third wave of COVID-19 outbreak, with the tally rising to 4189 as of 11 August 2020, which exceeds 3000, thus surpassing the numbers from the 2003 outbreak of severe acute respiratory syndrome (SARS) [4]. As the COVID-19 threat persists, maintaining public adherence to the precautionary measures is proving difficult. The adherence can depend upon a complex interplay of changing epidemiology, risk perceptions among the public, practicalities of exercising precautionary measures [5], government policies, and communications, as well as rapid exchange of trusted information and (mis)information in social media [6,7].

While human behavior plays a crucial role in controlling the spread of microbes and infections, little is known about the mechanism driving people to enact and adhere to precautionary behaviors that may essentially shape the spread or progression of COVID-19. Researchers in the United Kingdom recently conducted a cross-sectional survey to explore the underlying mechanism fostering hygienic practices according to the Capability, Opportunity, and Motivation Behavior (COM-B) model [8,9]. This model proposes that a person should have sufficient capability (i.e., physical and psychological capability to engage in the thought processes related to the activity concerned), motivation (i.e., conscious thought processes involving intention and planning) and opportunity (i.e., timing, resources, social and cultural norms) for a behavior to occur [8]. Physical and psychological capabilities are found to exert an indirect effect on adherence to precautionary measures for COVID-19 mediated by motivation, however, the items addressing both physical and psychological capabilities as a single construct might not adequately cover the psychological mechanism or drive of an individual’s adherence behavior [9]. Another survey conducted among adolescents in the United States suggested that the adolescents’ perceived risk of COVID-19 infection and social responsibility and trust would influence their adherence to preventive measures [10]. However, the role of other cognitive, adaptive, and maladaptive coping styles in response to COVID-19 have not been explored [11]. Indeed, COVID-19 “caution fatigue”, a new phenomenon whereby people become desensitized to warnings about COVID-19 due to feelings of prolonged stress, loneliness, and apathy, as well as disconfirming evidence, is a real-life example demonstrating how avoidance-based coping dominates and affects people’s collective engagement in combating the infection [12].

In the present study, we aimed to extend our understanding of how psychological factors affect adherence to precautionary measures for COVID-19 based on the hypotheses derived from Leventhal’s Common-Sense Model of Self-Regulation [13], which is one of the most widely used theoretical models to explain the processes of how a layperson’s perception of an illness threat guide coping strategies to deal with the threat [12]. The hypotheses outlined the relations between illness perceptions toward COVID-19 and behaviors in response to the outbreak of infection (i.e., adherence to COVID-19-related precautionary measures), which could be accounted for by a set of coping factors, including avoidance, problem-focused coping, seeking social support, and self-efficacy in following preventive measures. The hypothesized model would enable us to identify the component(s) that is/are most likely influencing adherence to the precautionary measures of COVID-19. Thus, the findings can inform the development of an evidence-based intervention fostering people’s adherence.

## 2. Materials and Methods

### 2.1. Participants and Procedures

The present study was part of a larger project, the COVID-IMPACT project (https://ucy.ac.cy/acthealthy/en/covid-19-impact-survey), which was an international online survey in 78 countries/regions worldwide exploring the behavioral and psychological impacts of COVID-19. This study recruited a convenience sample of Hong Kong citizens through online social media (e.g., Facebook, Instagram, Twitter, and WeChat), university mass emails and psychological associations between April and June 2020 (i.e., one month after the second wave of the COVID-19 pandemic in Hong Kong). Hong Kong Chinese residents aged 18 years or above with access to the Internet were eligible to participate in the study. An online, 20-min cross-sectional survey using a secured Google platform was used to collect data. Participants were informed of the study aims, risks, and benefits. Those who chose to participate provided informed consent. The survey was programmed as a forced response, such that all items on one page had to be completed in order to move to the next page, thereby avoiding missing data. Ethics approvals were obtained from the Cyprus National Bioethics Committee (ΕΕΒΚ ΕΠ 2020.01.60) and a University in Hong Kong (SBRE-19-593).

### 2.2. Measures

*Adherence to Precautionary Measures for COVID-19 Infection.* Three items (11-point Likert scale) were used to capture whether the participants adhered to precautionary measures in preventing COVID-19 transmission over the past, covering the following areas: physical distancing, limiting unnecessary travelling, and washing hands regularly with soap and water. A higher total score indicated better adherence. These items had adequate content validity (item content validity index, CVI = 0.95–0.98; scale CVI = 0.96) and internal consistency (Cronbach’s alpha, α = 0.85) in our sample.

*Illness Perceptions toward COVID-19.* Three subscales, including the Brief Illness Perception Questionnaire items assessing the perceived consequences, timeline, concern, and emotional responses toward COVID-19 (4 items, 10-point Likert scale) [14], the items assessing the perceived susceptibility (3 items, 6-point Likert scale) and severity of COVID-19 (3 items, 6-point Likert scale) according to the Health Belief Model were used to assess the participants’ overall illness perceptions toward COVID-19 [15]. For each subscale, a higher score indicated a stronger illness perception. For example, one question was “*How do you think COVID-19 will continue?*” The items had adequate content validity (item CVI = 0.92–0.99, scale CVI= 0.95) and internal consistency (α = 0.85–0.89) in our sample.

*Coping.* The Brief Coping Orientation to Problems Experienced (Brief COPE) inventory was adopted to measure situational and dispositional coping responses toward COVID-19 (4-point Likert Scale) [16]. In this study, three coping dimensions were assessed: seeking social support (use of emotional support, use of instrumental support, venting, and religion, 4 items), problem-solving (active coping and planning, 2 items) and avoidance (self-distraction, denial, substance use, behavioral disengagement, and self-blaming, 5 items). A higher score indicated a higher tendency to implement the corresponding coping strategy. The Brief COPE demonstrated adequate convergent validity [16], and the subscale items showed adequate internal consistencies in our sample (α = 0.79–0.84).

*Self-Efficacy.* Five items (7-point Likert scale) were used to assess the participant’s self-appraisal of his/her own competencies to plan and execute actions successfully in order to follow the precautionary measures of COVID-19. One example item was “*When facing difficulties in following the recommendations, I am certain that I will overcome them*.” A higher score indicates better self-efficacy. These items had adequate content validity (item CVI = 0.88–0.93, scale CVI = 0.96) and internal consistency (α = 0.84) in our sample.

Participants were also asked to report their age, gender, educational level, employment, family status, impacts of social isolation measures on daily activities and finance, and whether they and their family members were infected by COVID-19.

### 2.3. Statistical Analyses

**Figure 1:**
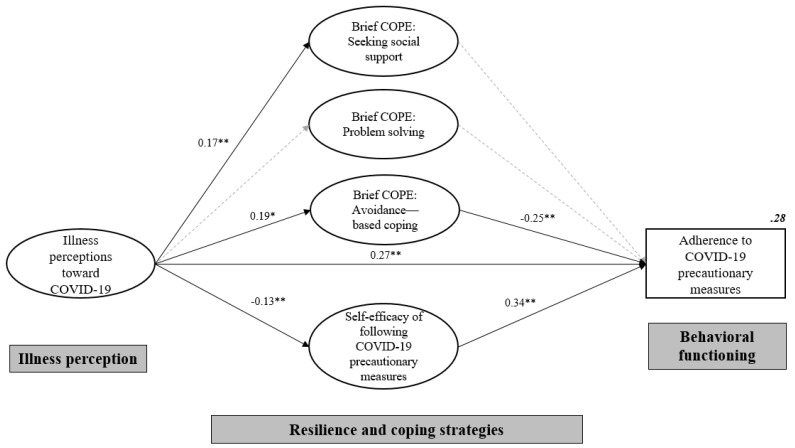
A structural equation model. Latent variables are represented by ellipses. Solid lines indicate significant paths of direct effects, dashed lines indicate non-significant paths of direct effects. For simplicity, the observed variables and covariance paths for all latent variables were not displayed. Model was adjusted for gender (male/female). All coefficients are standardized. Abbreviations: Brief COPE, Brief Coping Orientation to Problems Experienced Inventory. * *p* < 0.05, ** *p* < 0.01.

The SPSS software (Version 23.0; IBM Corp., Armonk, NY, USA, 2014) was used to perform descriptive statistics and correlational analysis, and the SPSS Analysis of Moments Structure software (Version 23.0; IBM Corp., Chicago, IL, USA, 2014) was used for structural equation modelling (SEM). We first examined the correlations between all the observed variables in the model. To interpret the correlation coefficients (*r*) (absolute *r*), we referred to Cohen’s criteria as follows: >0.10, small; >0.30, medium; >0.50, large [17]. Next, we performed the SEM analysis following the two-step approach as recommended by Anderson and Gerbing [18]. First, we conducted confirmatory factor analyses (CFA) for the following constructs, including illness perceptions toward COVID-19, seeking social support, problem solving, avoidance-based coping, and self-efficacy, to examine whether they were measured by the indicators with significant factor loadings. Second, based on the hypothesized path model, we examined an initial structural path model with adjustment for the socio-demographic and lifestyle characteristics as listed in Table 1. This model was then trimmed subsequently to obtain the final model (Figure 1) by dropping insignificant socio-demographics. The SEM parameters were estimated by the maximum likelihood method. Since chi-square test is sensitive to sample size, the following model fit indices were assessed, with values in parentheses indicating an acceptable fit: (1) the root mean square error of approximation (RMSEA ≤ 0.08), (2) the comparative fit index (CFI ≥ 0.90) and (3) the standardized root mean square residual (SRMR ≤ 0.1) [19]. All statistical tests were two-sided with level of significance set at 0.05.

## 3. Results

Table 1 presents the demographic characteristics of the participants. A total of 514 Hong Kong citizens (mean [SD] age, 32.75 [11.52] years) completed the survey. Owing to the convenience sampling method, information regarding the response rate was unavailable. In general, the sample consisted of mainly females (74.1%), holding an undergraduate degree or higher (81.9%) and working as non-health care professionals (88.3%). Around one-third of the sample reported that they had been staying at home and that their finances became worse since the imposed social isolation measures, and only one participant reported that he/she was infected by COVID-19.

Table 2 presents the means (SDs) and bivariate correlations among study variables. Adherence to precautionary measures for COVID-19 was significantly correlated with all items assessing self-efficacy of adhering to the precautionary measures (*r*s = 0.30–0.54, all *p*s < 0.01), problem-solving (*r*s = 0.15, all *p*s < 0.01), and avoidance-based coping (*r*s = −0.20–0.15, all *p*s < 0.01), respectively. The results of the CFA indicated that the measuring items corresponding to the latent constructs were all adequately fit (χ² = 431.55, *df* = 139, CFI = 0.9, SRMR = 0.07, RMSEA = 0.06).

Figure 1 shows the final SEM model. Fit statistics indicated that the SEM fitted the data well (χ² = 542.98, *df* = 169, CFI = 0.92, SRMR = 0.07, RMSEA = 0.06), although not all standardized path coefficients were significant. Illness perceptions toward COVID-19 showed direct effects on seeking social support (standardized path coefficient, β = 0.17 [95% CI, 0.56, 0.29], *p* = 0.004), avoidance-based coping (β = 0.19, [95% CI, 0.04, 0.33], *p* = 0.018), and self-efficacy (β = −0.13, [95% CI, −0.24, −0.01], *p* = 0.005). Avoidance-based coping (β = −0.25, [95% CI, −0.42, −0.08], *p* = 0.005) and self-efficacy (β = 0.34, [95% CI, 0.25, 0.43], *p* = 0.001) had direct effects on adherence to precautionary measures for COVID-19. The model explained 28% of the variance of the adherence to precautionary measures, adjusted for gender (male/female).

Table 3 summarizes the standardized direct, indirect, and total effects of illness perceptions toward COVID-19 on adherence to precautionary measures for COVID-19 based on the final SEM. Notably, people’s illness perceptions toward COVID-19 had significant direct effects on their adherence to precautionary measures (unstandardized β = 0.50, [95% CI, 0.28, 0.80], *p* = 0.001), and indirect effects through avoidance-based coping (β = −0.10 [95% CI, −0.26, −0.01], *p* = 0.016) and self-efficacy (β = −0.10, [95% CI, −0.18, −0.01], *p* = 0.025).

## 4. Discussion

This study examined the role of illness perceptions, coping, and self-efficacy on adherence to precautionary measures for COVID-19 among Hong Kong citizens one month after the second wave of the COVID-19 pandemic. Our SEM model provides useful insight into the psychological pathways of behaviors in controlling or preventing the spread of the COVID-19 infection, which can be important in informing risk management and public health strategies to address promotion and maintenance of desired protective behaviors for infection control. We found that illness perceptions toward COVID-19 significantly predicted adherence behaviors to precautionary measures. This finding implies that providing accurate, concise, and up-to-date information regarding the transmission, mechanism, symptom severity, and plausible treatment of COVID-19 can help increase public awareness of the hazards and risks of harm from COVID-19. Illness perception, which refers to the cognitive appraisal and personal understanding by people of a novel infectious disease [14], can largely influence not only their psychological and behavioral responses to the illness condition, but also their health outcomes [20].

When encountering the COVID-19 pandemic, unfamiliarity and uncertainty about the infectious disease and its catastrophic potential could lead to high anxiety and emotional reactions of the public [21], which might shape their acceptance of and adherence to risk mitigation. This phenomenon is reflected in our results, showing that avoidance-based coping partially and significantly accounted for the influence of Hong Kong people’s illness perceptions toward COVID-19 on their adherence to the precautionary measures. It is interesting to note that avoidance, but not problem-solving-based coping, has demonstrated a mediating role on adherence behaviors. This indicates that after the first and second waves of the COVID-19 pandemic, the Hong Kong people’s emotional reaction to the pandemic likely interfered with their capacity to formulate or engage in problem-solving-based coping strategies to manage barriers to their adherence to the precautionary measures. With high levels of uncertainty and emotional strain, the Hong Kong people might unwittingly choose and adopt avoidance-based coping (i.e., denial, self-blame, rumination, and hypervigilance by over-doing precautions to become desensitized over time) to manage the anxiety and distress concerning the infection risk and impact of the COVID-19 outbreak, thus negatively impacting their adherence behaviors. Such avoidance-based coping identified in this study is consistent with another survey conducted in Hong Kong in the initial stage of H1N1 pandemic, in which around 77% of Hong Kong citizens showed avoidance behavior (e.g., avoiding going out or touching potentially contaminated objects), which was not the advice or restriction policy of the government, and was likely attributed to their intense emotional reactions [22]. These findings are also consistent with another study reporting that affective processes consistently predicted the uptake of protective behaviors across several waves of H1N1 epidemics in Hong Kong [23].

Self-efficacy in executing and following the precautionary measures for COVID-19 was found to be another mediating factor in adherence to the measures in our SEM model. This is important, but already evidenced in social science literature showing that an individual’s perception of his/her competency to successfully perform the expected precautionary measures for COVID-19 affects his/her willingness and actual behavior to adhere to the measures [24]. More importantly, the simultaneous mediating effects co-produced by avoidance-based coping and self-efficacy might indicate an interactive relationship or effect of these two psychological constructs on affecting one’s perceived capability of adhering to precautionary measures for COVID-19. Hence, apart from improving the public’s self-efficacy in following the precautionary measures for COVID-19 through health education [25], an important but often neglected strategy is to address avoidance-based coping through effective interventions or resilience programs that are built from the framework of acceptance and commitment therapy, which is an empirically-based psychotherapy focusing on helping people to recognize their own avoidance and increasing their capacity to engage in values-based behavioral changes [21,26]. In addition, as suggested by the World Health Organization, public officials and health care providers should address the plausible emotional responses arising from the pandemic by using appropriate risk communication strategies at both individual and community levels. Such strategies include being responsive, honest, empathic, transparent, and consistent when delivering messages through trusted channels of communication, and monitoring infodemic and responding to rumors and misinformation with scientific evidence [27]. For any new regulatory measures that need to be imposed to address the COVID-19 pandemic, government officials should consider whether the public has adequate competency in adhering to the new measures, as well as their possible maladaptive psychological responses (e.g., avoidance) that may hinder the effect of these measures.

Our study findings should be interpreted with caution in view of the following study limitations. First, our data were cross-sectional and thus causal or temporal interpretations could not be made. Second, since the participants were self-selected into the study via social media platforms and university mass emails, the findings might be prone to selection bias. The respondents were primarily female (74.1%) and educated (81.9% graduated from universities), which might also limit the generalizability of our findings to the general populations. However, our findings were consistent with previous studies exploring the role of psychological factors in behavioral responses to the H1N1 pandemic [22,23], indicating its potential to apply in pandemics of different respiratory infectious diseases. Third, all data were self-reported and thus the participants’ responses might be subject to recall, response and/or social desirability biases.

Notably, the international COVID-IMPACT study commenced in April 2020. During that time, wearing surgical masks or face coverings was controversial, as there was no evidence or policy supporting the conclusion that wearing masks could reduce the spread of infection in the community. Therefore, with the recent increasing evidence on different effective precautionary measures, the questionnaire used for assessing adherence to these measures should include more items than those used in this study (i.e., with physical distancing, limiting unnecessary travelling, and washing hands regularly with soap and water only). Hence, the adequacy of the latent construct representing the adherence to precautionary measures for COVID-19 can be further improved with significant factor loadings. Further research to develop and validate an instrument serving to comprehensively assess the public’s adherence to precautionary measures of a novel infectious disease is also warranted. Finally, the percentage of the explained variance in adherence to precautionary measures of COVID-19 contributed by resilience and coping factors, together with the covariate (working as health care professionals), was only 28%. Further research should be conducted to test the model by including other contextual factors that affect individual’s adherence, such as social norms of perceiving illness threat, social inequality in accessing resources for adhering to precautionary measures, and the practicalities of exercising precautionary measures [5,28]. It has been recently suggested that the tendency of adhering to COVID-19 precautionary measures may imply the involvement of prosocialness in containing the spread of COVID-19, because such adherence behavior can bring societal and communal benefits (e.g., by helping protect your neighborhood) rather than benefiting oneself only [29,30]. The role of prosocialness in facilitating collective behavior, thereby encouraging better adherence to COVID-19 precautionary measures, or even the effectiveness of public health interventions that leverage prosocial messages to improve adherence, deserves future research. As political ideology and trust in government have often been found to affect vaccine attitudes and uptake among the public [31], future studies could explore whether individual political affiliation or the phenomenon of political polarization affects how the public perceives and responds to COVID-19 or other infectious disease outbreaks [32].

## 5. Conclusions

Understanding specific cognitive factors and coping affecting public adherence to precautionary measures for COVID-19 is paramount to mitigating the adversity and progress of the COVID-19 pandemic. As shown in our findings, illness perceptions toward COVID-19 contribute to adherence to COVID-19 precautionary measures, as well as through less avoidance-based coping and better self-efficacy in following the precautionary measures. Our findings suggest that only emphasizing the health hazards of a novel infectious disease is unlikely to adequately motivate and engage the public to make desired behavioral changes. An individual’s psychological capacity, in terms of avoidance-based coping and self-efficacy, should also be addressed.

## Figures and Tables

**Table 1:** Characteristics of the participants.

Characteristics	Respondents, No. (%) (*n* = 514)
Age, mean (SD) [range]	32.75 (11.52) [18–66]
Gender	-
Male	134 (25.9)
Female	380 (74.1)
Educational level	-
Primary school	44 (8.6)
Secondary school	49 (9.5)
University	239 (46.5)
Master/Postgraduate	150 (29.2)
Doctorate	32 (6.2)
Employment status	-
Working full time	326 (63.4)
Working part time	72 (14.0)
Unemployed	103 (20.0)
Others (retired/on leave)	13 (2.5)
Working as health care professional	-
No	444 (88.3)
Yes	59 (11.7)
Marital status	-
Single	233 (45.3)
In a relationship/engaged	108 (21.0)
Married	163 (31.7)
Others (divorced/widowed/separated)	10 (2.0)
Having children	-
No	393 (76.5)
Yes	121 (23.5)
Living situation	-
Living alone	38 (7.4)
Living with both parents	231 (44.9)
Living with one of the parents	34 (6.6)
Living with own family	179 (34.8)
Living with friends or roommates	32 (6.2)
Since the social isolation measures began, how frequent you needed to leave your house?	-
No	173 (33.7)
Once only	49 (9.5)
A couple of times	150 (29.2)
More than three times per week	142 (27.6)
Since the social isolation measures began, have your financial situation changed?	-
Have got better	38 (7.4)
Stay the same	322 (62.6)
Have got worse	154 (30.0)
Have you been infected by COVID-19?	-
Yes	1 (0.2)
No	507 (98.6)
I am not sure or have had symptoms but not diagnosed	6 (1.2)
Have your partner being infected by COVID-19?	-
Yes	1 (0.2)
No	475 (92.4)
I am not sure or have had symptoms but not diagnosed	6 (1.2)
Have your significant other being infected by COVID-19?	-
Yes	3 (0.6)
No	506 (98.4)
I am not sure or have had symptoms but not diagnosed	5 (1.0)

Abbreviations: COVID-19, coronavirus disease 2019.

**Table 2:** Means and correlations for all study variables.

Variable (No.)		Variable No., Correlation																			
	Mean (SD) [Range]	1	2	3	4	5	6	7	8	9	10	11	12	13	14	15	16	17	18	19	20
Adherence (1)	26.73 (3.37) [7–30]	1	0.21 ^b^	0.10 ^a^	0.16 ^b^	0.33 ^b^	0.30 ^b^	0.35 ^b^	0.46 ^b^	0.54 ^b^	0.10 ^a^	0.16 ^b^	0.08	0.05	0.15 ^b^	0.15 ^b^	0.15 ^b^	−0.16 ^b^	−0.05	−0.20 ^b^	−0.04
Illness perceptions toward COVID-19																					
Illness perceptions in terms of consequences, timeline, concern, and emotional responses (2)	26.84 (5.66) [7–40]	-	1	0.38 ^b^	0.40 ^b^	−0.04	−0.11 ^a^	−0.06	0.08	0.08	0.16 ^b^	0.11 ^a^	0.10 ^a^	−0.04	−0.07	−0.04	0.20 ^b^	0.15 ^b^	0.15 ^b^	0.06	0.08
Perceived susceptibility (3)	9.23 (2.99) [3–17]	-	-	1	0.40 ^b^	−0.13 ^b^	−0.16 ^b^	−0.14 ^b^	−0.04	−0.05	0.11 ^a^	0.07	0.08	0.04	−0.06	0.01	0.05	0.09 ^a^	0.10 ^a^	0.07	0.10 ^a^
Perceived severity (4)	14.55 (3.02) [3–18]	-	-	-	1	0.01	−0.02	−0.01	0.10 ^a^	0.08	0.12 ^b^	0.12 ^b^	0.11 ^a^	−0.03	0.06	0.12 ^b^	0.11 ^b^	−0.03	−0.02	−0.07	0.09
Self-efficacy																					
I have the skills to get through this difficult situation. (5)	5.95 (1.05) [1–7]	-	-	-	-	1	0.86 ^b^	0.79 ^b^	0.60 ^b^	0.63 ^b^	−0.02	0.03	0.02	0.09 ^a^	0.17 ^b^	0.15 ^b^	0.01	−0.09 ^a^	−0.10 ^a^	−0.18 ^b^	−0.09 ^a^
I can deal with this difficult situation. (6)	5.95 (1.00) [1–7]	-	-	-	-	-	1	0.84 ^b^	0.62 ^b^	0.66 ^b^	−0.04	0.01	0.02	0.10 ^a^	0.19 ^b^	0.14 ^b^	−0.01	−0.12 ^b^	−0.13 ^b^	−0.19 ^b^	−0.10 ^a^
When facing difficulties in following the recommendations, I am certain that I will overcome them. (7)	5.91 (1.00) [2–7]	-	-	-	-	-	-	1	0.67 ^b^	0.73 ^b^	0.02	0.06	0.05	0.08	0.20 ^b^	0.19 ^b^	0.03	−0.13 ^b^	−0.16 ^b^	−0.19 ^b^	−0.08
Compared to other people, I can follow these recommendations pretty well. (8)	5.98 (1.03) [1–7]	-	-	-	-	-	-	-	1	0.84 ^b^	0.08	0.10 ^a^	0.06	0.04	0.21 ^b^	0.24 ^b^	0.13 ^b^	−0.11 ^a^	−0.10 ^a^	−0.18 ^b^	−0.03
Even when things get tough, I can follow these recommendations quite well. (9)	5.90 (0.99) [2–7]	-	-	-	-	-	-	-	-	1	0.10 ^a^	0.12 ^b^	0.06	0.07	0.23 ^b^	0.22 ^b^	0.09 ^a^	−0.15 ^b^	−0.08	−0.22 ^b^	−0.07
Seeking social support																					
Use of emotional support (10)	4.75 (1.59) [2–8]	-	-	-	-	-	-	-	-	-	1	0.77 ^b^	0.57 ^b^	0.23 ^b^	0.33 ^b^	0.34 ^b^	0.42 ^b^	0.12 ^b^	0.12 ^b^	0.24 ^b^	0.36 ^b^
Use of instrumental support (11)	5.28 (1.52) [2–8]	-	-	-	-	-	-	-	-	-	-	1	0.57 ^b^	0.25 ^b^	0.38 ^b^	0.40 ^b^	0.41 ^b^	0.10 ^a^	0.14 ^b^	0.15 ^b^	0.29 ^b^
Venting (12)	5.23 (1.45) [2–8]	-	-	-	-	-	-	-	-	-	-	-	1	0.12 ^b^	0.32 ^b^	0.35 ^b^	0.36 ^b^	0.09 ^a^	0.03	0.16 ^b^	0.32 ^b^
Religion (13)	4.06 (1.92) [2–8]	-	-	-	-	-	-	-	-	-	-	-	-	1	0.19 ^b^	0.21 ^b^	0.10 ^a^	0.02	0.06	0.03	0.12 ^b^
Problem-solving																					
Active coping (14)	5.78 (1.44) [2–8]	-	-	-	-	-	-	-	-	-	-	-	-	-	1	0.64 ^b^	0.39 ^b^	−0.03	0.04	−0.10 ^a^	0.12 ^b^
Planning (15)	5.90 (10.34) [2–8]	-	-	-	-	-	-	-	-	-	-	-	-	-	-	1	0.28 ^b^	−0.04	0.05	−0.04	0.21 ^b^
Avoidance																					
Self-distraction (16)	5.19 (1.54) [2–8]	-	-	-	-	-	-	-	-	-	-	-	-	-	-	-	1	0.16 ^b^	0.07	0.11 ^a^	0.24 ^b^
Denial (17)	2.84 (1.16) [2–7]	-	-	-	-	-	-	-	-	-	-	-	-	-	-	-	-	1	0.12 ^b^	0.39 ^b^	0.24 ^b^
Substance use (18)	2.69 (1.34) [2–8]	-	-	-	-	-	-	-	-	-	-	-	-	-	-	-	-	-	1	0.12 ^b^	0.09 ^a^
Behavioral disengagement (19)	3.40 (1.26) [2–8]	-	-	-	-	-	-	-	-	-	-	-	-	-	-	-	-	-	-	1	0.48 ^b^
Self-blaming (20)	4.33 (1.49) [2–8]	-	-	-	-	-	-	-	-	-	-	-	-	-	-	-	-	-	-	-	1

^a^*p* < 0.05, calculated using 2-tailed bivariate correlations. ^b^ *p* < 0.01, calculated using 2-tailed bivariate correlations. For each variable, a high score indicates a greater extent of the underlying trait being measured. Abbreviations: COVID-19: coronavirus disease 2019.

**Table 3:** Direct, indirect, and total effects of illness perceptions toward COVID-19 on adherence to COVID-19 precautionary measures.

Paths	Unstandardized Path Coefficient, β Estimate (95% CI)	Standardized Path Coefficient, β Estimate (95% CI)	*p* Value
Direct effect			
IP → Adherence	0.50 (0.28, 0.80)	0.27 (0.15, 0.38)	0.001
Indirect effects	-	-	-
IP → Seeking social support → Adherence	0.04 (−0.01, 0.12)	-	0.063
IP → Problem-solving → Adherence	0.00 (−0.05, −0.05)	-	0.978
IP → Avoidance-based coping → Adherence	−0.10 (−0.26, −0.01)	-	0.016
IP → Self-efficacy of following COVID-19 precautionary measures → Adherence	−0.10 (−0.18, −0.01)	-	0.025
Total effect	-	-	-
IP → Adherence	0.43 (0.21, 0.70)	0.23 (0.12, 0.34)	<0.001

Abbreviations: COVID-19, coronavirus disease 2019; IP, illness perceptions toward COVID-19.
